# Aerobic Capacity, Physical Activity and Metabolic Risk Factors in Firefighters Compared with Police Officers and Sedentary Clerks

**DOI:** 10.1371/journal.pone.0133113

**Published:** 2015-07-17

**Authors:** Roman Leischik, Peter Foshag, Markus Strauß, Henning Littwitz, Pankaj Garg, Birgit Dworrak, Marc Horlitz

**Affiliations:** 1 Department of Cardiology, Faculty of Health, School of Medicine, University Witten/Herdecke, Hagen, Germany; 2 University of Leeds, Leeds Institute of Cardiovascular and Metabolic Medicine (LICAMM), Leeds, United Kingdom; 3 Department of Cardiology, Faculty of Health, School of Medicine, University Witten/Herdecke, Cologne, Germany; Indiana University Richard M. Fairbanks School of Public Health, UNITED STATES

## Abstract

**Background:**

This study examined the association between the physical work environment and physiological performance measures, physical activity levels and metabolic parameters among German civil servants. A main focus in this study was to examine the group differences rather than measuring the absolute values in an occupational group.

**Methods:**

We prospectively examined 198 male German civil servants (97 firefighters [FFs], 55 police officers [POs] and 46 sedentary clerks [SCs]). For each parameter, the groups were compared using a linear regression adjusted for age.

**Results:**

The 97 FFs showed a similar maximal aerobic power (VO_2max_ l/min) of 3.17±0.44 l/min compared with the POs, who had a maximal aerobic power of 3.13±0.62 l/min (estimated difference, POs vs. FFs: 0.05, CI: -0.12-0.23, p=0.553). The maximal aerobic power of the FFs was slightly higher than that of the SCs, who had a maximal aerobic power of 2.85±0.52 l/min (-0.21, CI: -0.39-0.04, p=0.018 vs. FFs). The average physical activity (in metabolic equivalents [METS]/week) of the FFs was 3818.8±2843.5, whereas those of the POs and SCs were 2838.2±2871.9 (-808.2, CI: 1757.6-141.2, p=0.095) and 2212.2±2292.8 (vs. FFs: -1417.1, CI: -2302-531.88, p=0.002; vs. POs: -2974.4, CI: -1611.2-393.5, p=0.232), respectively. For the FFs, the average body fat percentage was 17.7%±6.2, whereas it was 21.4%±5.6 for the POs (vs. FFs: 2.75, CI: 0.92-4.59, p=0.004) and 20.8%±6.5 for the SCs (vs. FFs: 1.98, CI: -0.28-4.25, p=0.086; vs. POs: -0.77, CI: 3.15-1.61, p=0.523). The average waist circumference was 89.8 cm±10.0 for the FFs, 97.8 cm±12.4 (5.63, CI: 2.10-9.15, p=0.002) for the POs, and 97.3±11.7 (vs. FFs: -4.89, CI: 1.24-8.55, p=0.009; vs. POs: -0.73, CI: -5.21-3.74, p=0.747) for the SCs.

**Conclusions:**

The FFs showed significantly higher physical activity levels compared with the SCs. The PO group had the highest cardiovascular risk of all of the groups because it included more participants with metabolic syndrome; furthermore, the POs had an average of 2.75% higher body fat, lower HDL cholesterol values and higher waist circumferences compared with the FFs and higher LDL cholesterol values compared with the SCs. Our data indicate that sedentary occupations appear to be linked to obesity and metabolic syndrome in middle-aged men.

## Introduction

Heart disease causes 45% of the deaths among US firefighters (FFs) while they are on duty [1]. Police officers (POs) have a poorer health prognosis and more metabolic disorders than the general population [2, 3]. The majority of prospective studies have found that occupational sitting was associated with a higher risk of diabetes mellitus and mortality [4]. Healy et al. described deleterious associations of prolonged sedentary time with cardio-metabolic and inflammatory biomarkers in US adults [5]. In the 1950s, employment that required hard physical work had positive influences on mortality [6]. However, in the present day, work-related physical activity is not related to body mass index and obesity [7], and “white collar” workers seem to have less cardiovascular diseases compared to “blue collar” workers [8]. Additionally, middle-aged production workers in Korea have poorer overall physical condition than middle-aged office workers [9]. Thus, occupational physical activity seems to not be currently related to health benefits compared to leisure time activity [10]. Generally, physical inactivity is thought to be responsible for up to 25% of all breast and colorectal cancer cases, up to 27% of diabetes mellitus cases, and up to 30% of ischemic coronary heart disease cases [11]. The association between higher physical activity levels and positive changes in metabolic risk parameters is a known phenomenon [12, 13]. This correlation between fitness and health is supported by findings that professional endurance athletes live longer compared with the general population [14].

Different groups of civil servants have different health issues [15]. For some people who are employed full-time (e.g., sedentary clerks [SCs]), sitting in an occupational context can consume an average of 26–45.2 h/week [16]. Office workers who sit for large proportion of their working day also report sitting for longer outside work [17]. Social circumstances at work show an inverse association between social class, as assessed by grade of employment, and mortality from a wide range of diseases [15]. In Germany, all civil servants have a similar secure and stable social situation and belongs to the social “middle class”.

This study sought to examine the association between the physical work environment and physiological performance measures, physical activity levels and metabolic parameters among three groups of German civil servants with different occupational physical activity profiles. Aerobic capacity, physical activity and metabolic functioning have never been compared prospectively in consecutively recruited male German officials.

## Subjects and Methods

### Subjects

Career firefighters from Wuppertal, Hagen, and Witten; federal police officers from Düsseldorf and Bonn; and sedentary office workers from Hagen and Witten were invited to participate in this study via the internet, social media and local corporate distribution after they responded to an official request. A main focus in this study was to examine the group differences rather than measuring the absolute values in an occupational group. Maximal aerobic power (VO_2max_ l/min) [18–20] and aerobic capacity [21] (relative VO_2_ [ml/kg^-1^·min^-1^] at the aerobic threshold) were estimated using spiroergometry. Physical activity was measured using a definition of “vigorous METS” (jogging, cycling, swimming, football, martial arts sports, and strength training) according to the Compendium of Physical Activities guidelines [22] to estimate the differences in energy consumption among the different groups of civil servants. A definition of “total METS” was used to represent all of the physical activities during a day (including slow walking, sleeping, sitting-according to Ainsworth et al. [23]). “Global METS” indicates METS reached during the exercise test (spiroergometry) and are comparable with VO_2max_. Cardiovascular risks and metabolism were assessed using cholesterol, triglyceride, glycated hemoglobin and homocysteine levels. A definition of metabolic syndrome published by Alberti et al. [24] was used.

All of the examinations were performed from 1.1.2014 to 06.15.2014 at the Sports Medicine Center in Hagen (Research Sector Prevention, Public Health and Sports Medicine, University Witten-Herdecke).

### Experimental protocol

Spiroergometry [18, 19, 21] was performed in the following manner: A stress test was conducted continuously after successful gas and volume calibration beginning at 50 watts and continuously increasing by 25 watts every 2 min (ramp test). The test ended when the subject could no longer maintain the predefined cadence of 80/min or if the subject was subjectively exhausted and there was no further increase in VO_2max_ after 20 sec. The spiroergometric analyses were conducted as previously described [18, 21]. The ventilator aerobic threshold (VAT) was defined as the first non-linear increase in the ventilatory equivalent for oxygen without a simultaneous increase of the ventilatory equivalent for CO_2_. The respiratory compensation point (RCP) was defined as the simultaneous non-linear increase of both ventilatory equivalents according to previously described recommendations [18, 19, 21]. Body weight and body composition were determined using a Tanita BC-418MA segmental body composition analyzer [25]. The subjects were instructed to wear only comfortable shorts without any other clothing. A special questionnaire based on Ainsworth et al. [22] was used to calculate the global METS values that were reached weekly.

### Ethical statement

All of the participants provided verbal and written consent to voluntarily participate in the testing and to allow the use of their data in this study. All of the data were anonymized. This study was approved as a doctoral dissertation by the Dissertation Audit Committee of the University Witten/Herdecke. Additionally, University Witten/Herdecke ethics committee approval was granted in 2013 (no. 121/2013). This study did not introduce any pharmaceutical interventions or changes in the clinical course of the participants. In cases of evident clinical illness, a medical report was sent to the participant’s family doctor.

### Statistical analysis

The data are described separately for the three groups. The anthropometric parameters, clinical characteristics, and physical activity and cardiorespiratory fitness parameters are described using the means, standard deviations, minimums and maximums. The differences among the groups were estimated using a linear regression adjusted for age because most of the analyzed parameters were directly age-related; 95% confidence intervals (CIs) are also reported. All of the statistical tests were two-sided with a significance level of 0.05. Because of the exploratory nature of the study, the p-values were not adjusted for multiple tests. Stata/IC 13.1 for Windows was used for the statistical analysis. A dichotomous variable was created for each level of the categorical variable profession (PO, FF, and SC), and the different profession levels were compared (contrasted) with a specified reference level. Because we wanted to estimate all pairwise differences among the three categories, the POs and SCs were compared with the reference category of the FFs, and the SCs were compared with the POs (reference PO).

## Results

In this prospective study, all 198 consecutively recruited male participants (97 FFs, 55 POs and 46 SCs) were included in the study. All of the participants belonged to the same social stratum (“middle class”). All of the participants were working as officials at police departments, fire departments or municipal authorities. The SCs were civil servants who worked in tax offices or municipal administrations and performed desk work while in a sitting position. The sample sizes of the two smallest groups (n = 46 (SC) and n = 55 (PO)) were sufficient to achieve a statistical power of 0.8 for an effect size of 0.4 (significance level 0.05, two-sided t-test).

The analysis of the interaction term “age and profession” did not result in a different interpretation of the results.

The clinical characteristics (heart rate, arterial blood pressure) and anthropometric data (Table 1) did not differ among the groups. None of the volunteers had a history of stroke or coronary disease, and all of the participants were considered healthy. The waist circumference and body fat percentage differed among the groups. The FFs had a smaller average waist circumference than the POs and SCs: the average waist circumference of the FFs was 89.8 cm±10.0, whereas it was 97.8 cm±12.4 for the POs (estimated difference vs. FFs: 5.63 (CI: 2.10–9.15, p = 0.002) and 4.89 for the SCs (CI: 1.24–8.55, p = 0.009 vs. FFs and vs. POs, ns). The body fat percentage of the FFs was significantly lower than that of the POs: 17.7%±6.2 vs. the POs: 21.4%±5.6 (2.75, CI: 0.92–4.59, p = 0.004) and not significantly different from that of the SCs (1.98, CI: -0.29–4.25, p = 0.086). The POs showed greater muscle mass than the FFs (67.1 kg±6.9 (2.75, CI: 0.24–5.27), p = 0.032) and the SCs (65.3 kg ±6.4 (-4.48, CI: -7.27–1.70), p = 0.002).

**Table 1:** Anthropometry: description and estimated group differences (linear regression adjusted for age).

	FFs	POs	SCs	POs vs. FFs	SCs vs. FFs	SCs vs. POs
	n			Estimated difference		
	Mean			(95%-CI)		
	SD			p-value		
Weight in kg	n = 97	n = 55	n = 46	6.69	0.02	-6.67
	85.9	93.6	87.1	(2.47–10.90)	(-4.47–4.51)	(-11.85–1.49)
	(11.5)	(13.2)	(13.4)	p = 0.002	p = 0.994	p = 0.012
Height in cm	n = 97	n = 55	n = 46	1.04	-0.21	-1.25
	182.2	182.8	181.5	(-1.10–3.18)	(-2.22–1.80)	(-3.65–1.14)
	(6.3)	(6.8)	(5.4)	p = 0.340	p = 0.835	p = 0.304
BMI	n = 97	n = 55	n = 46	1.65	0.08	-1.57
	25.9	28.0	26.4	(0.57–2.74)	(-1.22–1.39)	(-3.02–0.12)
	(3.2)	(3.2)	(4.1)	p = 0.003	p = 0.899	p = 0.034
BSA	n = 97	n = 55	n = 46	0.09	-0.00	-0.09
	2.08	2.18	2.09	(0.03–0.14)	(-0.06–0.06)	(-0.16–0.02)
	(0.16)	(0.18)	(0.17)	p = 0.003	p = 0.950	p = 0.011
Muscle mass in kg	n = 97	n = 55	n = 46	2.75	-1.73	-4.48
	67.1	69.7	65.3	(0.24–5.27)	(-4.12–0.66)	(-7.27–1.70)
	(6.9)	(7.8)	(6.4)	p = 0.032	p = 0.155	p = 0.002
% body fat	n = 97	n = 55	n = 46	2.75	1.98	-0.77
	17.7	21.4	20.8	(0.92–4.59)	(-0.29–4.25)	(-3.15–1.61)
	(6.2)	(5.6)	(6.5)	p = 0.004	p = 0.086	p = 0.523
Waist circumference in cm	n = 97	n = 55	n = 46	5.63	4.89	-0.73
	89.8	97.8	97.3	(2.10–9.15)	(1.24–8.55)	(-5.21–3.74)
	(10.0)	(12.4)	(11.7)	p = 0.002	p = 0.009	p = 0.747
Heart rate_rest_	n = 97	n = 55	n = 46	0.86	1.92	1.07
	67.6	68.9	70.1	(-3.08–4.79)	(-2.59–6.43)	(-3.66–5.80)
	(12.0)	(11.4)	(12.5)	p = 0.669	p = 0.401	p = 0.657
Systolic blood pressure_rest_	n = 97	n = 55	n = 46	1.04	2.26	1.22
	126.4	127.9	129.1	(-3.07–5.15)	(-1.71–6.24)	(-3.51–5.94)
	(9.8)	(12.4)	(11.7)	p = 0.617	p = 0.263	p = 0.612
Diastolic blood pressure_rest_	n = 97	n = 55	n = 46	1.01	1.90	0.89
	84.1	85.7	86.6	(-2.14–4.17)	(-0.99–4.79)	(-2.83–4.60)
	(7.4)	(10.2)	(8.7)	p = 0.527	p = 0.197	p = 0.639
Cigarettes/day	n = 97	n = 55	n = 46	-0.30	-1.13	-0.83
	2.57	2.52	1.72	(-2.45–1.85)	(-3.16–0.90)	(-3.08–1.43)
	(6.26)	(5.98)	(5.53)	p = 0.782	p = 0.275	p = 0.469

BMI = body mass index; BSA = body surface area; CI = confidence interval (95%); FFs = firefighters; POs = police officers; n = number of participants; SCs = sedentary clerks; sd = standard deviation; mean = mean value.

The frequency of metabolic syndrome (FFs 12.4%, POs 36.4%, SCs 30.4%) in all participants was dependent on the occupational group (logistic regressions analysis, p = 039). The age-adjusted pairwise odds ratio (95% CI) for POs vs FFs was 3.0 (1.3–71, p = 0.011), for SCs vs FFs was 2.0 (0.7–5.2, p = 0.183) and for SCs vs POs was 0.6 (0.3–1.6, p = 0.351).

Smoking behavior was independent of occupational group (logistic regression p = 0.544), with no significant differences observed between the groups (FFs 17.5%, POs 18.2%, SCs 10.9%).

### Physical activity

Physical activity (Table 2) was estimated in METS, as recommended by Ainsworth et al. [22]. To address the possible errors and pitfalls [26–28], we used two approaches for analysis: the METS total represents the sum of all athletic team activities, such as football, basketball, volleyball, and handball; individual sport activities, such as inline skating, tennis, squash, gymnastics, and strength training; moderate activities, such as walking and Nordic walking; and vigorous activities, such as jogging, swimming and cycling. Sleeping, sitting, car driving and standing were not taken into account. The second approach included only activities with vigorous METS values (strength training, jogging, swimming, and cycling). The FFs were more active than the SCs because on-duty FFs have more opportunities for training when they are waiting for assignments; the POs showed physical activity levels similar to those of the SCs. The FFs and POs had the option of participating in corporate sports programs. Data regarding the vigorous METS activities are shown in Table 2.

**Table 2:** Physical activity: descriptions and estimated group differences (linear regression adjusted for age).

	FFs (1)	POs (2)	SCs (3)	POs vs. FFs	SCs vs. FFs	SCs vs. POs
	n			Estimated difference		
	Mean			(95%-CI)		
	(SD)			p-value		
Leisure time sports h/week	n = 97	n = 55	n = 46	-0.72	-1.60	-0.67
	6.0	4.74	4.03	(-2.16–0.73)	(-2.79–0.41)	(-2.17–0.66)
	(3.6)	(4.30)	(3.02)	p = 0.329	p = 0.008	p = 0.292
Corporate sports h/week	n = 97	n = 53	n = 46	-0.81	-1.21	-0.41
	1.50	0.58	0.15	(-1.19–0.42)	(-1.58–0.85)	(-0.72–0.10)
	(1.52)	(0.93)	(0.63)	p = 0.000	p = 0.000	p = 0.010
Strength training h/week	n = 92	n = 55	n = 46	-0.32	-0.79	-0.47
	1.73	1.20	0.71	(-0.95–0.30)	(-1.34–0.24)	(-1.08–0.14)
	(1.81)	(1.88)	(1.27)	p = 0.307	p = 0.005	p = 0.133
Strength training METs/week	n = 92	n = 55	n = 46	-121.37	-343.48	-222.11
	804	594	363.1	(-427.66–184.92)	(-612.84–74.12)	(-528.48–84.26)
	(836)	(925)	(652.1)	p = 0.435	p = 0.013	p = 0.154
Martial arts activities h/week	n = 91	n = 55	n = 46	0.03	-0.04	-0.07
	0.27	0.27	0.20	(-0.35–0.41)	(-0.37–0.30)	(-0.46–0.33)
	(1.02)	(1.05)	(0.98)	p = 0.878	p = 0.832	p = 0.741
Martial arts activities METs	n = 91	n = 55	n = 46	19.13	-2.50	-21.63
	246	233.0	208.4	(-320.04–358.30)	(-339.49–334.5)	(-403.21–359.96)
	(930)	(913.0)	(1031)	p = 0.912	p = 0.988	p = 0.911
Swimming h/week	n = 92	n = 55	n = 46	-0.16	-0.07	0.09
	0.33	0.14	0.23	(-0.32–0.01)	(-0.26–0.12)	(-0.11–0.30)
	(0.65)	(0.38)	(0.59)	p = 0.039	p = 0.473	p = 0.358
Swimming METS	n = 92	n = 55	n = 46	-61.65	-31.52	30.14
	151.5	77.9	106.8	(-140.07–16.76)	(-123.22–60.19)	(-70.13–130.40)
	(300.4)	(211.4)	(280.8)	p = 0.123	p = 0.499	p = 0.554
Football h/week	n = 91	n = 55	n = 46	-0.04	-0.20	-0.15
	0.46	0.36	0.20	(-0.42–0.34)	(-0.48–0.09)	(-0.45–0.14)
	(1.08)	(0.96)	(0.54)	p = 0.830	p = 0.178	p = 0.299
Football METS	n = 91	n = 55	n = 46	9.10	-101.10	-110.20
	261.3	231.4	117.3	(-220.09–238.29)	(-265.62–63.42)	(-296.05–75.66)
	(636.9)	(621.5)	(328.7)	p = 0.938	p = 0.227	p = 0.244
Jogging h/week	n = 92	n = 55	n = 46	-0.14	-0.29	-0.15
	1.77	1.45	1.29	(-0.78–0.50)	(-1.14–0.56)	(-0.99–0.69)
	(2.00)	(1.74)	(2.43)	p = 0.662	p = 0.498	p = 0.723
Jogging METs	n = 92	n = 55	n = 46	-38.38	-226.43	-188.05
	977	900	703	(-427.2–350.4)	(-692.6–239.72)	(-651.2–275.1)
	(1161)	(1072)	(1280)	p = 0.846	p = 0.339	p = 0.424
Cycling h/week	n = 92	n = 55	n = 46	-1.30	-1.41	-0.11
	2.36	1.20	1.10	(-2.31–0.29)	(-2.28–0.54)	(-0.99–0.77)
	(3.10)	(2.69)	(1.75)	p = 0.012	p = 0.002	p = 0.806
Cycling METs	n = 92	n = 55	n = 46	-821.18	-822.43	-98.04
	1514	802	713	(-1473.65–168.70)	(-1361.18–283.68)	(-674.16–475.43)
	(1967)	(1754)	(1139)	p = 0.014	p = 0.003	p = 0.733
Vigorous METs /week	n = 90	n = 55	n = 46	-985.22	-1598.79	-613.57
	3953	2838	2212	(-1940.56–29.88)	(-2476.51–721.06)	(-1617.40–390.26)
	(2688)	(2872)	(2293)	p = 0.043	p = 0.000	p = 0.229

**Fig 1:**
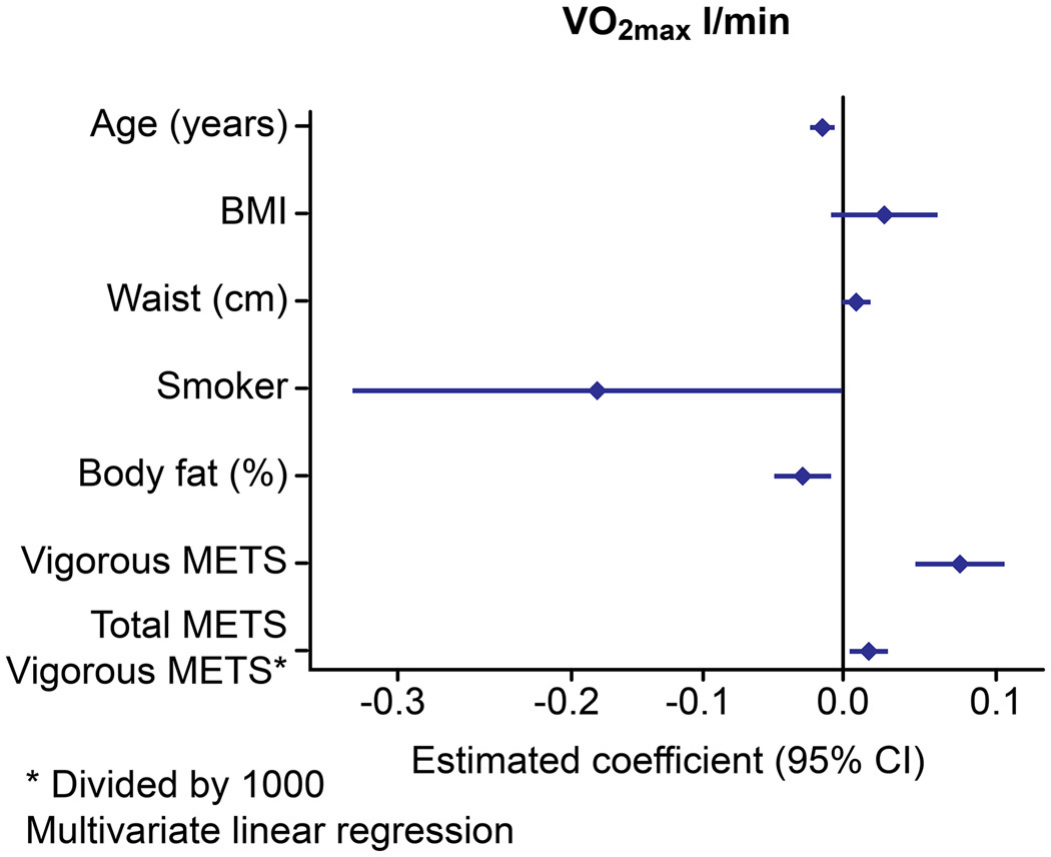
Multivariate analysis of the factors influencing absolute VO_2max_.

**Fig 2:**
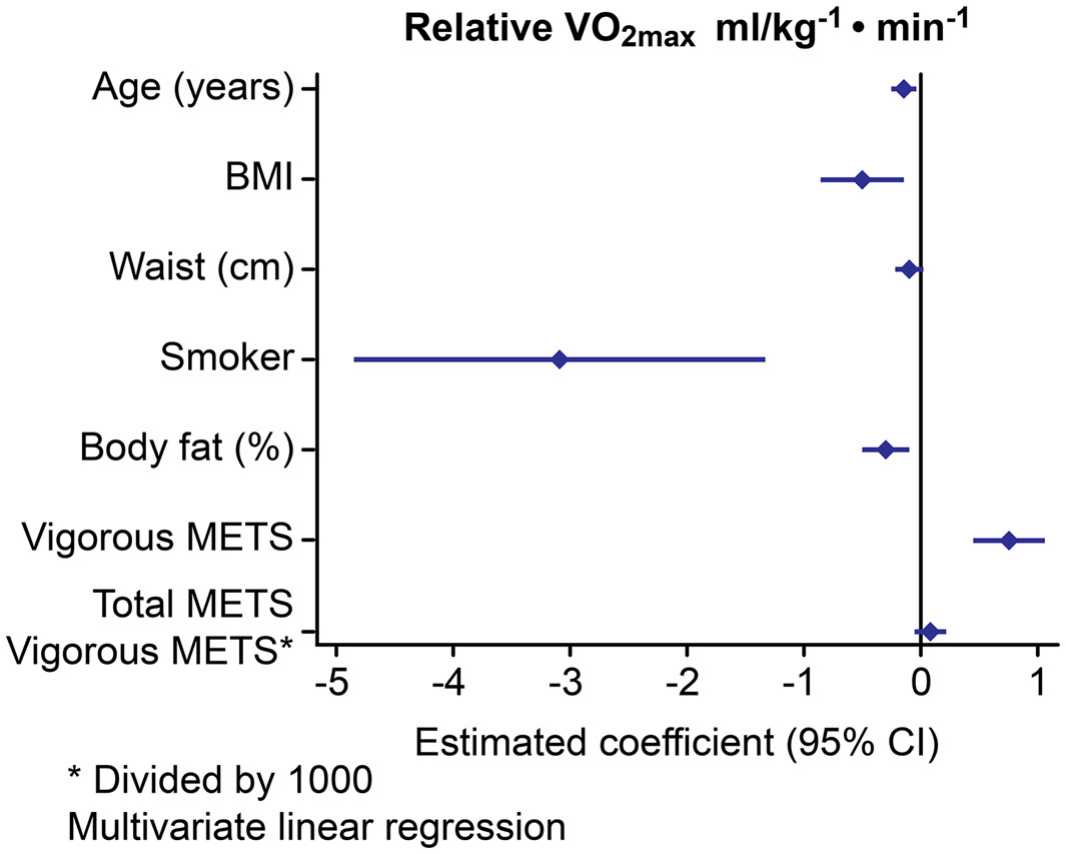
Multivariate analysis of the factors influencing relative VO_2max_.

Our results indicate that VO_2max_ and relVO_2max_ are dependent on reported vigorous METS and independent of global METS, which includes additional walking, sitting, sleeping, homework and golfing (Figs 1 and 2).

### Cardiorespiratory fitness and performance

In the FFs, the VO_2max_ as an absolute value was significantly higher (3.17 1±0.44) compared with that of the SCs: 2.85±0.5 2 (-0.21, CI: -0.39–0.04, p = 0.018) but was similar to that of the POs: 3.13 l±0.62, (0.05, CI: -0.12–0.23, p = 0.553). Multivariate analysis showed that the absolute VO_2max_ (Fig 1) and relativeVO_2max_ (Fig 2) were positively influenced by vigorous physical activity and negatively influenced by smoking. The relative VO_2max_ ml/kg^-1^·min^-1^ was similar for all of the groups. The aerobic capacity (VO_2AT_) did not differ among the groups. The maximal power (W_max_) was significantly higher in the FFs and POs than in the SCs (-32.07, CI: -47.9–16.2, p = 0.004 vs. the FFs and -29.9, CI: -47.3–12.6, p = 0.001 vs. the POs). After weight adjustment (W_max_/kg), the FFs were stronger than the POs (-0.26, CI: -0.47–0.05, p = 0.014) and the SCs (3.03±0.74 (-0.36, CI: 0.60–0.12), p = 0.004). These results are shown in Table 3. The maximal METS values during exercise were similar for all three groups.

**Table 3:** Spiroergometry: description and estimated group differences (linear regression adjusted for age).

	FFs	POs	SCs	POs vs. FFs	SCs vs. FFs	SCs vs. POs
	n			Estimated difference		
	Mean			(95%-CI)		
	SD			p-value		
HR_max_	n = 97	n = 55	n = 46	1.28	-2.97	-4.26
	175.0	172.4	167.7	(-3.40–5.97)	(-8.17–2.22)	(-9.92–1.41)
	(14.4)	(16.2)	(17.5)	p = 0.589	p = 0.260	p = 0.140
HR_RCP_	n = 97	n = 55	n = 46	4.59	0.32	-4.28
	130.7	133.9	129.4	(-2.47–11.66)	(-7.24–7.88)	(-12.73–4.18)
	(20.6)	(21.2)	(22.0)	p = 0.201	p = 0.934	p = 0.320
Absolute VO_2max_	n = 97	n = 55	n = 46	0.05	-0.21	-0.27
	3.17	3.13	2.85	(-0.12–0.23)	(-0.39–0.04)	(-0.48–0.05)
	(0.44)	(0.62)	(0.52)	p = 0.553	p = 0.018	p = 0.015
Absolute VO_2AT_	n = 97	n = 55	n = 46	0.10	-0.07	-0.17
	1.56	1.64	1.47	(-0.06–0.27)	(-0.24–0.11)	(-0.36–0.02)
	(0.53)	(0.49)	(0.47)	p = 0.227	p = 0.466	p = 0.079
Absolute VO_2RCP_	n = 97	n = 55	n = 46	0.12	-0.08	-0.20
	2.14	2.24	2.04	(-0.12–0.35)	(-0.31–0.14)	(-0.47–0.07)
	(0.60)	(0.73)	(0.65)	p = 0.324	p = 0.470	p = 0.148
Relative VO_2max_	n = 97	n = 55	n = 46	-1.71	-1.55	0.16
	37.3	34.1	34.1	(-3.96–0.53)	(-4.15–1.04)	(-2.82–3.15)
	(6.3)	(8.0)	(8.1)	p = 0.134	p = 0.240	p = 0.915
Relative VO_2AT_	n = 97	n = 55	n = 46	-0.32	0.16	0.48
	18.7	17.9	18.3	(-2.32–1.68)	(-2.90–3.23)	(-2.66–3.62)
	(6.3)	(5.8)	(9.4)	p = 0.756	p = 0.916	p = 0.763
Relative VO_2RCP_	n = 97	n = 55	n = 46	-0.47	-0.81	-0.35
	25.4	24.4	24.0	(-3.19–2.25)	(-3.62–1.99)	(-3.65–2.96)
	(7.3)	(8.6)	(8.2)	p = 0.736	p = 0.568	p = 0.836
W_max_	n = 97	n = 55	n = 46	-2.14	-32.07	-29.93
	299.1	288.5	257.7	(-16.71–12.42)	(-47.91–16.24)	(-47.31–12.55)
	(43.3)	(48.6)	(46.9)	p = 0.772	p = 0.000	p = 0.001
W_max/kg_	n = 97	n = 55	n = 46	-0.26	-0.36	-0.10
	3.53	3.14	3.03	(-0.47–0.05)	(-0.60–0.12)	(-0.36–0.16)
	(0.64)	(0.68)	(0.74)	p = 0.014	p = 0.004	p = 0.469
METS, absolute	n = 97	n = 55	n = 45	0.04	2.59	2.56
	10.7	9.7	12.2	(-1.21–1.28)	(-3.70–8.89)	(-2.72–7.84)
	(1.8)	(2.3)	(17.9)	p = 0.953	p = 0.418	p = 0.341

HR_max_ = maximum heart rate; HR_RCP_ = heart rate at respiratory compensation point; METS = metabolic equivalents; n = number of participants; RCP = respiratory compensation point; relative = absolute value in ml/kg^-1^ ·min^-1^; VO_2max_ = maximum oxygen uptake; VO_2AT_ = oxygen uptake at the aerobic threshold; VO_2RCP_ = oxygen uptake at the respiratory compensation point; W_max_ = maximum watts.

### Metabolic parameters and metabolic syndrome

The metabolic parameters are shown in Table 4. The HDL cholesterol values of the POs were lower than those of the FFs, and the homocysteine values were higher in the POs compared with the FFs. There was a tendency for the POs to have higher values for triglycerides, uric acid, and LDL cholesterol, but this tendency was not significant.

**Table 4:** Blood analysis of metabolic risk factors and estimated group differences (linear regression adjusted for age).

	FFs	POs	SCs	POs vs. FFs	SCs vs. FFs	SCs vs. POs
	n			Estimated difference		
	Mean			(95%-CI)		
	(SD)			p-value		
HbA1c %	n = 97	n = 55	n = 46	-0.04	-0.10	-0.06
	5.4	5.5	5.4	(-0.15–0.06)	(-0.26–0.06)	(-0.24–0.13)
	(0.3)	(0.4)	(0.6)	p = 0.429	p = 0.234	p = 0.552
Glucose mg/dl	n = 97	n = 55	n = 46	5.52	-3.29	-8.81
	65.5	72.9	64.3	(-1.11–12.15)	(-10.42–3.83)	(-17.13–0.49)
	(17.6)	(20.9)	(22.1)	p = 0.102	p = 0.363	p = 0.038
Cholesterol mg/dl	n = 97	n = 55	n = 46	-0.80	-0.80	0.00
	199.1	205.9	206.6	(-12.74–11.14)	(-10.68–9.08)	(-12.53–12.53)
	(34.2)	(40.4)	(29.9)	p = 0.895	p = 0.873	p = 1.000
Triglycerides mg/dl	n = 97	n = 55	n = 46	28.40	3.87	-24.54
	142.2	185.1	162.1	(-9.04–65.85)	(-25.55–33.28)	(-69.19–20.12)
	(75.1)	(145.4)	(91.4)	p = 0.136	p = 0.796	p = 0.280
HDL cholesterol mg/dl	n = 97	n = 55	n = 46	-5.16	1.36	6.52
	55.5	49.4	55.8	(-10.01–0.31)	(-3.51–6.22)	(0.64–12.40)
	(12.7)	(15.3)	(14.8)	p = 0.037	p = 0.583	p = 0.030
LDL cholesterol mg/dl	n = 96	n = 55	n = 46	7.24	-3.92	-11.16
	115.6	129.1	118.6	(-3.84–18.32)	(-13.09–5.25)	(-22.48–0.17)
	(31.6)	(37.7)	(23.6)	p = 0.199	p = 0.400	p = 0.054
Creatinine	n = 97	n = 55	n = 46	-0.00	-0.06	-0.06
	0.94	0.94	0.89	(-0.05–0.05)	(-0.11–0.01)	(-0.11–0.00)
	(0.16)	(0.15)	(0.13)	p = 0.959	p = 0.026	p = 0.045
Uric acid mg/dl	n = 97	n = 55	n = 46	0.04	0.02	-0.02
	5.6	5.7	5.7	(-0.33–0.41)	(-0.35–0.38)	(-0.42–0.38)
	(1.1)	(1.1)	(0.9)	p = 0.834	p = 0.924	p = 0.917
Urea mg/dl	n = 97	n = 55	n = 46	0.20	-1.22	-1.42
	33.5	33.5	32.1	(-2.22–2.62)	(-3.88–1.44)	(-4.17–1.32)
	(8.3)	(6.7)	(7.2)	p = 0.870	p = 0.366	p = 0.308
Homocysteine mmol/l	n = 97	n = 55	n = 46	2.68	1.39	-1.29
	14.1	17.0	15.8	(0.88–4.49)	(0.11–2.68)	(-3.29–0.71)
	(3.1)	(6.1)	(4.1)	p = 0.004	p = 0.034	p = 0.204

Metabolic syndrome [29] is defined as a minimum of 3 of the following factors: waist circumference ≥94, systolic blood pressure ≥130 mmHg, fasting glucose ≥100 mg/dl, triglycerides ≥150 mg/dl, and HDL cholesterol ≤40 mg/dl. The requirements for metabolic syndrome were found in 12 out of 98 FFs (13.4%), 20 out of 45 POs (36.4%), 14 out of 46 SCs (30.43%) and 46 out of the total group of 198 participants (23.2%). Lipoprotein(a) >30 mg/dl [30] was found in 36 FFs (37.1%), 14 POs (25.5%), 17 SCs (37.0%) and 67 of the total group of 198 participants (33.8%). Homocysteine >15 mg/dl was reported in 28 FFs (28.9%), 30 POs (54.5%), 21 SCs (45.7%) and 79 of the total group of 198 participants (39%).

## Discussion

This study shows that German civil servants such as POs and SCs are more likely to have metabolic syndrome and a higher waist circumference compared with German FFs. The FFs and POs displayed significantly higher maximal power (W_max_) compared with the SCs. After weight adjustment (W_max_/kg), the FFs were stronger than the POs and the SCs. The small clinical differences in aerobic capacity (measured with spiroergometry as relative VO_2_ ml/kg^-1^·min^-1^ at the aerobic threshold) between the FFs and SCs are somewhat surprising. There were no significant differences among the FF, PO and SC groups other than minimal, clinically irrelevant deviations in the absolute maximal oxygen uptake (VO_2max_) between the FFs and SCs. Physical activity, which was determined using questionnaires, suggested a difference in aerobic capacity that favored the FFs and POs; however, questionnaires might not be sufficient for determining objective differences among individual professional groups [31]. Questionnaire responses depend on the respondents’ perception, encoding, storage and retrieval of information about their previous physical activity; furthermore, the answers depend on the subject’s age and the context of the questioning [31]. Although the use of questionnaires and the comparison of VO_2max_ [32] or other parameters of cardiorespiratory fitness (e.g., METS or maximal exercise [33, 34]) have been common practice for many years, validation tests have indicated that these assessment methods have some vulnerabilities [31]. For example, one study found no association between firefighters’ self-perception of their level of fitness and their aerobic capacity as measured by either the step test or submaximal treadmill test [35]. The literature suggests that results related to physical activity are useful for investigating the individual situations of specific populations within a study, but comparisons among studies and sites are highly questionable. Therefore, spiroergometry, which offers information about absolute and relative VO_2max_ and possibly aerobic capacity, VO_2maxAT_, must be considered the gold standard [27, 36, 37]. It is well known that questionnaires can yield falsely high or unreliable values concerning physical activity [26, 27]; nonetheless, they are commonly used for gathering information about large cohorts and for evaluating prognoses [31, 33].

All of the participating German FFs were career FFs undergoing continuous supervision of their health and fitness. The relatively good findings related to obesity, waist circumference and cardiorespiratory fitness in the German FFs can be explained by the official and corporate care they receive. In the USA, 72% of FFs are volunteers, and only 28% are career FFs [38]. This fact might explain the high prevalence of coronary disease and metabolic disorders among US FFs [38]. However, metabolic syndrome and obesity have also been found in career FFs in the US, indicating that these disorders represent both a national problem and a problem with the quality of corporate guidelines [39–41].

In the current study, we examined three German civil servant occupations. A comparison of the results with the data in general population can be interesting. Jackson et al. [42] described a body mass index (BMI) of 25.9±3.3 and a prevalence of 11.7% current smokers in men (aged 48±10.3 years). Using a treadmill test, an average METS (absolute METS in our study) of 12.3±2.3 was reached. Lakoski et al. [43] reported in a gender mixed cohort of 20,329 Caucasians a mean cardiorespiratory level of 10.7±1.9 METS in mean aged 40–49 years. The BMI in this group (28.1 ±3.2) was similar to the results of our study. Additionally, the previous study reported systolic blood pressure in mixed genders of 120.2 (±14.0) and diastolic blood pressure of 81.6(±9.7). Additionally, the prevalence of current smokers was reported to be 13.7%.

### Cardiorespiratory fitness in the studied occupations

Physical activity is recognized as one of the most important protectors against cardiovascular diseases and cancer worldwide [41, 44–46]. Cardiorespiratory fitness protects against obesity and diabetes [47–49]. FFs require a high maximal cardiorespiratory fitness to perform in difficult circumstances while outfitted with full emergency equipment [50]. Firefighting involves a unique set of stressors: strenuous muscular work; stair climbing; carrying and using heavy tools; working in dangerous environments and under extreme temperatures; exposure to toxic smoke; and the psychological stress of an emergency situation. All of these factors require extremely good cardiorespiratory fitness, endurance and resistance. The estimated relVO_2max_ (VO_2max_ ml/kg^-1^·min^-1^) proposed for firefighting ranges from 33.6 to 46 ml/kg^-1^·min^-1^ [50]. The physiological demands for firefighter candidates are an average of 38 ml/kg^-1^·min^-1^ [51]. However, examining relVO_2max_ ranges is complicated due to the different equipment for VO_2max_ measurements [50]. In our study, we used one of the most validated types of equipment for this purpose. The German FFs showed an acceptable average VO_2max_ value, but this value was lower than that of recreational triathletes [52]. Emergency situations and carrying full emergency equipment may require a higher VO_2max_; some of the well-trained FFs in our study had a maximal value of 54 ml/kg^-1^·min^-1^. The lowest value was 26 ml/kg^-1^·min^-1^. However, the maximal power (W_max_) was significantly higher in FFs than in SCs (-32.07, CI: -47.9–16.2, p = 0.004). After weight adjustment (W_max_/kg), the FFs were stronger than the SCs (3.53±0.64 vs. 3.03±0.74; -0.36, CI: 0.60–0.12, p = 0,004) and the POs (3.53±0.64 vs. 3.14±0.68; -0.26, CI:-0-47-0.05, p = 0.014). Worldwide, POs belong to a community with a high prevalence of obesity [53, 54] and cardiovascular risk factors [2] and a lower life expectancy [3] compared with the general population. It is unknown why POs are more obese and have a comparatively high rate of metabolic syndrome. Some possible explanations include environmental stress, rotating shifts and poor nutrition. It is unfortunate that a group whose members are selected into the field based on remarkable physical fitness fails to maintain this level of fitness and succumbs to lifestyle diseases that are largely preventable [55]. The mean maximal oxygen uptake in POs is 42.1±8.9 ml/kg^-1^·min^-1^ [56]. In one study, Finnish POs [57] showed a VO_2max_ of 3.4±0.8 l (relVO_2max_ 42.8±10.1) at an early age, but 15 years later, at a mean age of 49 years (42–61), they had a VO_2max_ of 3.3±0.7 (relVO_2max_ 38.4±8.3 ml/kg^-1^·min^-1^). In our study, the VO_2max_ of the POs was similar (3.13 l±0.62). Pollock et al. [58] reported a relVO_2max_ of 40.7 ± 4.5 ml/kg^-1^·min^-1^ in young POs (21 to 35 years). In another study, young police volunteers performing a two-day POPAT test [59] showed excellent cardiorespiratory fitness with an average VO_2max_ of 3.75±0.52 (relVO_2max_ 44.1±6.6 ml/kg^-1^·min^-1^). However, it is not possible to accurately compare the values of these previous studies because of the different circumstances and the different equipment used.

The physical demands on on-duty policemen can be high at any given time, for example, if they are at rest and interventions suddenly become necessary. However, accelerometer measurements suggest that male POs engage in less job-related physical activity than secretaries and that they appear to be more active on their off-duty days than during their work hours [60]. However, if this is not the case, they tend to become obese. Additionally, there is a correlation between activity levels and the areas of police duty, which can require different demands. In general, policemen who are more stressed tend to be less active [60]. The results of our study support the suggestion that there is a need to intensify training programs and provide diet/nutrition training courses to improve the obesity and metabolic risks associated with police work.

Office workers from Malaysia showed a relVO_2max_ of 24±3.8 ml/kg^-1^ · min^-1^ [61]. Korean office workers demonstrated a relVO_2max_ of 32.4±5.4 ml/kg^-1^ min^-1^ [9]. Duque et al. [62] described a VO_2max_ of 2.82±0.4 l/min and relVO_2max_ of 40.5±5.5 ml/kg^-1^ min^-1^ in healthy men (aged 39.3±7.8). Krausharr et al. [63] examined 83 obese employees of a German electronics manufacturer. These participants had body weights up to 92.6±13.1 kg and a VO_2max_ of 32.2±8.01 ml/kg^-1^·min^-1^. Spiroergometry was performed using equipment similar to that used in our study. The values observed in these previous studies are similar to those observed in SCs in the present study. Specifically, a maximal aerobic power of 2.82 l/min in healthy controls was reported by Duque et al. [62], which is equivalent to the VO_2max_ of the SCs in our study. It can be assumed that the estimated VO_2max_ and relVO_2max_ values of our SCs are similar to those of healthy volunteers from a “normal” population.

### Metabolic risks and obesity

Firefighting is widely regarded as a hazardous occupation. Unfortunately, in this profession, avoidable risk factors for coronary disease are relatively common (at least in the USA). Soteriades et al. [38] reported that FFs died of coronary heart disease (CHD) on-duty had a twofold higher relative risk of tobacco abuse, a threefold higher risk of obesity and a twofold higher risk for elevated cholesterol levels compared with CHD-caused retirements. Additionally, metabolic syndrome is inversely related to cardiorespiratory fitness in career FFs [39]. In a study that included 957 FFs, triglycerides were elevated in 28.5%, HDL cholesterol was reduced in 40.8%, and blood glucose was >100 mg/dl in 26.1% of the participants. Furthermore, obesity appears to be common among US FFs, worsening during follow-up to an average BMI of 30 [64]. Wilkinson et al. [65] reported that 82.5% of FFs are overweight (BMI 25.0–29.9 kg/m^2^) or obese (BMI >30.0 kg/m^2^). The negative metabolic situation of American and Indian POs [53, 54] is a known phenomenon, likely because the real occupational physical effort is not as high as expected and is comparable to that of a sedentary occupation [60]. Compared with the general population of the USA, a higher percentage of POs are obese (40.5% vs. 32.1%), have metabolic syndrome (26.7% vs. 18.7%), and have higher serum total cholesterol levels (200.8 mg/dl vs. 193.2 mg/dl) [2]. Thayyil et al. [55] reported a prevalence of metabolic syndrome in 16.8% of their study population of 900 POs. Hartley et al. [66] prospectively investigated 288 consecutive POs from a New York police department (mean age 41.7±7) and considered metabolic syndrome present in individuals who had three or more of the following properties: 1) abdominal obesity >102 cm, 2) hypertension: systolic blood pressure at rest >130 mmHg, 3) HDL cholesterol <40 mg/dl, 4) triglycerides >150 mg/dl, and 5) fasting glucose >100 mg/dl. Metabolic syndrome was diagnosed in 33% of these POs, whereas it was diagnosed in 31% of the POs in our study. In our study, 35% of the POs had a waist circumference >102 cm (Hartley et al. [66] reported a similar value of 38.9% in POs). Thus, it can be assumed that the high rate of metabolic syndrome among POs is a global problem.

Normally, mortality from CHD is higher among manual than non-manual occupational classes [67]. Clear data about sedentary civil servants that can be compared with the data for the SCs in our study are difficult to find and can only be indirectly extracted from studies in Britain [68] and the Netherlands [69, 70]. It can be suggested that the current BMI value (our study 2014) has a tendency to be higher among German civil servants (26.4±4.1) compared with British civil servants (Marmot et al 1974 (24.5±0.09)) [68]. In total, 43.5% of German SCs were overweight, but only 13% were obese. Additionally, appears that smoking behavior prevalence differs between these populations (28.8% of the British civil servants were current smokers, but only 20% of the German SCs in our study were current smokers). Moreover, cholesterol levels do not appear to be clinically different between British civil servants (201 mg/dl±1.72) and German SCs (206.6 ± 29.9). The values found in our study were lower than those reported for civil servants in the Netherlands (1953–1954) [69], who showed high cholesterol levels (266±50 mg/dl) but a similar BMI compared with the British civil servants (24.5±3.5).

### Limitations of the study

The results for self-reported activity among our study may be affected by the self-images of the members of these occupational groups. This effect can be assumed for the POs in our study. Many studies have focused on self-reported physical activity measures, which are affected by recall bias [31]. The voluntary character of the participation should be considered because it might influence the absolute values/results. However, the differences between the groups should not be affected. A main focus in this study was to examine the group differences and not to measure the absolute values in an occupational group. In a democratic country, there are no other ethical alternatives to voluntary participation.

## Conclusions

The results of this German CLERK study suggest that POs, whose cardiorespiratory fitness levels are similar to those of SCs and who exhibit an incidence of obesity even higher than that of the SCs, are unhealthy.

The German FFs clearly show a lower incidence of metabolic syndrome compared with the POs and SCs. Moreover, they are less frequently obese (10.3%) than their US colleagues (up to 40%) [64].

FFs must display cardiorespiratory fitness, but to a significantly lower degree than that of recreational triathletes, for example[52]. Furthermore, the present study shows a wide range of cardiorespiratory fitness among the FFs (from 26 to 54 ml/kg^-1^·min^-1^).

The consequences are obvious: there is a need for diet programs and corporate training for POs. For FFs, the training skills and techniques to increase aerobic capacity and aerobic power must be improved. Finally, SC training must incorporate diet programs and physical activity (44% of SCs are overweight). The effects of interventions on lipid levels, blood pressure are inconsistent, but BMI and frequency of cardiac events can be significantly reduced [71]. Simple stairclimbing programs can enhance cardiovascular fitness[72]. Worksite interventions have the potential to counter the increasing burden of overweight and obesity, large waist circumference, and lack of physical inactivity [73].

## Supporting Information

S1 Table Multivariate results—linear regression analysis. (DOCX)
