# Correction: Aerobic Capacity, Physical Activity and Metabolic Risk Factors in Firefighters Compared with Police Officers and Sedentary Clerks

**DOI:** 10.1371/journal.pone.0136224

**Published:** 2015-08-14

**Authors:** Roman Leischik, Peter Foshag, Markus Strauß, Henning Littwitz, Pankaj Garg, Birgit Dworrak, Marc Horlitz

There are errors in the third sentence of the Results section of the Abstract. The correct sentence is: The average physical activity (in metabolic equivalents [METS]/week) of the FFs was 3953±2688, whereas those of the POs was 2838±2872 (vs. FFs: -985, CI: -1941-30, p = 0.043) and of the SCs 2212±2293 (vs. FFs: -1598.8, CI: -2477-721, p = 0.000; vs. POs: -613.6, CI: -1617.4–390.3, p = 0.229), respectively.

## References

[1] Leischik R, Foshag P, Strauß M, Littwitz H, Garg P, Dworrak B, et al (2015) Aerobic Capacity, Physical Activity and Metabolic Risk Factors in Firefighters Compared with Police Officers and Sedentary Clerks. PLoS ONE 10(7): e0133113 doi:10.1371/journal.pone.0133113 26186442 10.1371/journal.pone.0133113 PMC4506022

