# Supplementary material for: Aerobic Capacity, Physical Activity and Metabolic Risk Factors in Firefighters Compared with Police Officers and Sedentary Clerks
Source: PLoS One. 2015 Jul 17;10(7):e0133113. doi: 10.1371/journal.pone.0133113 (PMC4506022; doi:10.1371/journal.pone.0133113)
Supplement: S1 Table — (DOCX) [file pone.0133113.s001.docx]

**S1 Aerobic Capacity, Physical Activity and Metabolic Risk Factors in Firefighters Compared with Police Officers and Sedentary Clerks**

**Table. Multivariate results - linear regression analysis.**

|  | Linear regression, estimated coefficients (95% CI), p-value and R^2^ | | | | |
| --- | --- | --- | --- | --- | --- |
|  | Age | PO vs. FF | SC vs. FF | SC vs. PO | R^2^ |
| Weight | 0.22 (0.05-0.39)  p=0.013 | 6.69 (2.47-10.90)  p=0.002 | 0.02 (-4.47-4.51)  p=0.994 | -6.67 (-11.85--1.49)  p=0.012 | 0.09 |
| Height in cm | -0.08 (-0.18-0.01)  p=0.073 | 1.04 (-1.10-3.18)  p=0.340 | -0.21 (-2.22-1.80)  p=0.835 | -1.25 (-3.65-1.14)  p=0.304 | 0.02 |
| BMI | 0.09 (0.04-0.14)  p=0.000 | 1.65 (0.57-2.74)  p=0.003 | 0.08 (-1.22-1.39)  p=0.899 | -1.57 (-3.02--0.12)  p=0.034 | 0.11 |
| BSA (m^2^) | 0.00 (-0.00-0.00)  p=0.083 | 0.09 (0.03-0.14)  p=0.003 | -0.00 (-0.06-0.06)  p=0.950 | -0.09 (-0.16--0.02)  p=0.011 | 0.07 |
| Muscle mass in kg | -0.02 (-0.11-0.08)  p=0.743 | 2.75 (0.24-5.27)  p=0.032 | -1.73 (-4.12-0.66)  p=0.155 | -4.48 (-7.27--1.70)  p=0.002 | 0.05 |
| % body fat | 0.21 (0.12-0.30)  p=0.000 | 2.75 (0.92-4.59)  p=0.004 | 1.98 (-0.29-4.25)  p=0.086 | -0.77 (-3.15-1.61)  p=0.523 | 0.17 |
| Waist in cm | 0.48 (0.33-0.62)  p=0.000 | 5.63 (2.10-9.15)  p=0.002 | 4.89 (1.24-8.55)  p=0.009 | -0.73 (-5.21-3.74)  p=0.747 | 0.24 |
| HFRest | 0.11 (-0.08-0.30)  p=0.266 | 0.86 (-3.08-4.79)  p=0.669 | 1.92 (-2.59-6.43)  p=0.401 | 1.07 (-3.66-5.80)  p=0.657 | 0.01 |
| BPsRest | 0.08 (-0.08-0.25)  p=0.310 | 1.04 (-3.07-5.15)  p=0.617 | 2.26 (-1.71-6.24)  p=0.263 | 1.22 (-3.51-5.94)  p=0.612 | 0.01 |
| BPdRest | 0.11 (-0.01-0.23)  p=0.063 | 1.01 (-2.14-4.17)  p=0.527 | 1.90 (-0.99-4.79)  p=0.197 | 0.89 (-2.83-4.60)  p=0.639 | 0.03 |
| Tobacco/day | 0.05 (-0.03-0.13)  p=0.208 | -0.35 (-2.48-1.78)  p=0.748 | -1.13 (-3.16-0.90)  p=0.275 | -0.78 (-3.02-1.46)  p=0.492 | 0.01 |
| Alcohol (week) | 0.00 (-0.02-0.02)  p=0.904 | 0.25 (-0.24-0.73)  p=0.318 | 0.31 (-0.27-0.89)  p=0.289 | 0.06 (-0.62-0.75)  p=0.857 | 0.01 |
| Sports h/week | -0.08 (-0.14--0.01)  p=0.022 | -0.93 (-2.32-0.46)  p=0.190 | -1.60 (-2.79--0.41)  p=0.009 | -0.67 (-2.08-0.73)  p=0.348 | 0.08 |
| Corporate sports h/week | -0.03 (-0.05--0.01)  p=0.011 | -0.82 (-1.20--0.43)  p=0.000 | -1.21 (-1.58--0.85)  p=0.000 | -0.40 (-0.71--0.09)  p=0.011 | 0.22 |
| Strength training h/week | -0.04 (-0.07--0.02)  p=0.002 | -0.32 (-0.95-0.30)  p=0.307 | -0.79 (-1.34--0.24)  p=0.005 | -0.47 (-1.08-0.14)  p=0.133 | 0.10 |
| Strength training METS/week | -18.38 (-30.30--6.46)  p=0.003 | -80.58 (-381.78-220.62)  p=0.598 | -302.31 (-566.40--38.21)  p=0.025 | -221.73 (-528.08-84.62)  p=0.155 | 0.08 |
| Martial arts sports h/week | -0.01 (-0.02-0.01)  p=0.347 | 0.03 (-0.35-0.41)  p=0.878 | -0.04 (-0.37-0.30)  p=0.832 | -0.07 (-0.46-0.33)  p=0.741 | 0.01 |
| Martial arts METS/week | -6.52 (-20.99-7.95)  p=0.375 | 33.79 (-295.04-362.63)  p=0.840 | 12.30 (-318.68-343.27)  p=0.942 | -21.50 (-403.01-360.02)  p=0.912 | 0.00 |
| Swimming h/week | -0.01 (-0.02-0.01)  p=0.336 | -0.16 (-0.32--0.01)  p=0.039 | -0.07 (-0.26-0.12)  p=0.473 | 0.09 (-0.11-0.30)  p=0.358 | 0.03 |
| Swimming METS/week | -2.48 (-7.54-2.59)  p=0.336 | -61.65 (-140.07-16.76)  p=0.123 | -31.52 (-123.22-60.19)  p=0.499 | 30.14 (-70.13-130.40)  p=0.554 | 0.02 |
| Football h/week | -0.01 (-0.03-0.01)  p=0.181 | -0.04 (-0.42-0.34)  p=0.830 | -0.20 (-0.48-0.09)  p=0.178 | -0.15 (-0.45-0.14)  p=0.299 | 0.03 |
| Football METS/week | -8.09 (-19.23-3.04)  p=0.153 | 9.10 (-220.09-238.29)  p=0.938 | -101.10 (-265.62-63.42)  p=0.227 | -110.20 (-296.05-75.66)  p=0.244 | 0.03 |
| Jogging h/week | -0.03 (-0.06--0.01)  p=0.016 | -0.14 (-0.78-0.50)  p=0.662 | -0.29 (-1.14-0.56)  p=0.498 | -0.15 (-0.99-0.69)  p=0.723 | 0.03 |
| Jogging METS/week | -19.14 (-34.14--4.14)  p=0.013 | 15.50 (-364.59-395.59)  p=0.936 | -172.01 (-630.57-286.54)  p=0.460 | -187.51 (-650.33-275.30)  p=0.425 | 0.03 |
| Cycling h/week | 0.03 (-0.01-0.07)  p=0.204 | -1.30 (-2.31--0.29)  p=0.012 | -1.41 (-2.28--0.54)  p=0.002 | -0.11 (-0.99-0.77)  p=0.806 | 0.06 |
| Cycling METS/week | 18.85 (-6.89-44.60)  p=0.150 | -724.39 (-1364.25-84.53)p=0.027 | -822.43 (-1361.18-283.68)p=0.003 | -98.04 (-671.69-475.61)p=0.736 | 0.05 |
| Vigorous METS | -35.86 (-75.91-4.38) p=0.081 | -808.22 (-1757.65-141.20) p=0.095 | -1417.07 (-2302.25-531.88)p=0.002 | -608.84 (-1611.21-393.52) p=0.232 | 0.07 |
| HR_max_ | -0.80 (-1.03--0.57)  p=0.000 | 1.28 (-3.40-5.97)  p=0.589 | -2.97 (-8.17-2.22)  p=0.260 | -4.26 (-9.92-1.41)  p=0.140 | 0.24 |
| HR_AT_ | -0.33 (-0.64--0.01)  p=0.044 | 4.91 (-1.52-11.34)  p=0.134 | 0.41 (-6.90-7.72)  p=0.912 | -4.50 (-11.36-2.36)  p=0.197 | 0.03 |
| HR_RCPr_ | -0.29 (-0.62-0.03)  p=0.076 | 4.59 (-2.47-11.66)  p=0.201 | 0.32 (-7.24-7.88)  p=0.934 | -4.28 (-12.73-4.18)  p=0.320 | 0.02 |
| abs. VO_2max_ | -0.02 (-0.03--0.01)  p=0.000 | 0.05 (-0.12-0.23)  p=0.553 | -0.21 (-0.39--0.04)  p=0.018 | -0.27 (-0.48--0.05)  p=0.015 | 0.16 |
| abs. VO_2AT_ | -0.01 (-0.01-0.00)  p=0.188 | 0.10 (-0.06-0.27)  p=0.227 | -0.07 (-0.24-0.11)  p=0.466 | -0.17 (-0.36-0.02)  p=0.079 | 0.02 |
| abs.VO_2RCPr_ | -0.00 (-0.01-0.01)  p=0.370 | 0.12 (-0.12-0.35)  p=0.324 | -0.08 (-0.31-0.14)  p=0.470 | -0.20 (-0.47-0.07)  p=0.148 | 0.02 |
| rel. VO_2max_ | -0.31 (-0.41--0.20)  p=0.000 | -1.71 (-3.96-0.53)  p=0.134 | -1.55 (-4.15-1.04)  p=0.240 | 0.16 (-2.82-3.15)  p=0.915 | 0.18 |
| rel. VO_2AT_ | -0.10 (-0.20--0.00)  p=0.049 | -0.32 (-2.32-1.68)  p=0.756 | 0.16 (-2.90-3.23)  p=0.916 | 0.48 (-2.66-3.62)  p=0.763 | 0.02 |
| rel.VO_2RCPr_ | -0.10 (-0.22-0.02)  p=0.098 | -0.47 (-3.19-2.25)  p=0.736 | -0.81 (-3.62-1.99)  p=0.568 | -0.35 (-3.65-2.96)  p=0.836 | 0.02 |
| %VO_2maxAT_ | 0.18 (-0.03-0.40)  p=0.096 | 1.31 (-3.40-6.02)  p=0.585 | -0.15 (-4.86-4.55)  p=0.949 | -1.46 (-6.76-3.84)  p=0.588 | 0.02 |
| VEmax | -0.65 (-1.03--0.27)  p=0.001 | 7.68 (0.26-15.10)  p=0.043 | 2.15 (-6.48-10.79)  p=0.624 | -5.53 (-14.81-3.75)  p=0.241 | 0.08 |
| W_max_ | -1.77 (-2.42--1.12)  p=0.000 | -2.14 (-16.71-12.42)  p=0.772 | -32.07 (-47.91--16.24)  p=0.000 | -29.93 (-47.31--12.55)  p=0.001 | 0.22 |
| W_RCPr_ | -0.24 (-1.06-0.57)  p=0.555 | 5.20 (-14.73-25.14)  p=0.607 | -16.51 (-34.71-1.68)  p=0.075 | -21.72 (-43.52-0.09)  p=0.051 | 0.02 |
| W_max/kg_ | -0.03 (-0.04--0.02)  p=0.000 | -0.26 (-0.47--0.05)  p=0.014 | -0.36 (-0.60--0.12)  p=0.004 | -0.10 (-0.36-0.16)  p=0.469 | 0.22 |
| HR RCP | -0.26 (-0.58-0.07)  p=0.127 | 2.95 (-4.48-10.39)  p=0.434 | -0.35 (-8.16-7.46)  p=0.930 | -3.30 (-11.62-5.02)  p=0.434 | 0.01 |
| BPs_Wmax_ | 0.20 (-0.10-0.50)  p=0.186 | -1.48 (-7.86-4.91)  p=0.649 | -3.13 (-9.86-3.59)  p=0.359 | -1.66 (-8.84-5.53)  p=0.650 | 0.01 |
| BP_Wmax_ | 0.24 (0.06-0.42)  p=0.008 | 2.52 (-0.73-5.76)  p=0.128 | 0.86 (-4.23-5.96)  p=0.739 | -1.65 (-7.15-3.84)  p=0.553 | 0.05 |
| HR_Wmax_ | -0.82 (-1.05--0.59)  p=0.000 | 1.00 (-3.69-5.70)  p=0.674 | -3.02 (-8.18-2.13)  p=0.248 | -4.03 (-9.66-1.61)  p=0.160 | 0.25 |
| METS, absolute | -0.20 (-0.42-0.02)  p=0.078 | 0.04 (-1.21-1.28)  p=0.953 | 2.59 (-3.70-8.89)  p=0.418 | 2.56 (-2.72-7.84)  p=0.341 | 0.05 |
| HbA1c, % | 0.02 (0.01-0.02)  p=0.000 | -0.04 (-0.15-0.06)  p=0.429 | -0.10 (-0.26-0.06)  p=0.234 | -0.06 (-0.24-0.13)  p=0.552 | 0.14 |
| HbA1C mmol/mol | 0.16 (0.08-0.25)  p=0.000 | 0.01 (-1.37-1.39)  p=0.987 | -0.68 (-2.61-1.25)  p=0.487 | -0.69 (-2.69-1.31)  p=0.495 | 0.10 |
| Glucose mg/dl | 0.39 (0.10-0.67)  p=0.008 | 5.52 (-1.11-12.15)  p=0.102 | -3.29 (-10.42-3.83)  p=0.363 | -8.81 (-17.13--0.49)  p=0.038 | 0.06 |
| Cholesterol mg/dl | 1.58 (1.12-2.03)  p=0.000 | -0.80 (-12.74-11.14)  p=0.895 | -0.80 (-10.68-9.08)  p=0.873 | 0.00 (-12.53-12.53)  p=1.000 | 0.17 |
| Triglycerides mg/dl | 3.03 (1.53-4.53)  p=0.000 | 28.40 (-9.04-65.85)  p=0.136 | 3.87 (-25.55-33.28)  p=0.796 | -24.54 (-69.19-20.12)  p=0.280 | 0.10 |
| HDL cholesterol mg/dl | -0.19 (-0.40-0.03)  p=0.092 | -5.16 (-10.01--0.31)  p=0.037 | 1.36 (-3.51-6.22)  p=0.583 | 6.52 (0.64-12.40)  p=0.030 | 0.05 |
| LDL cholesterol mg/dl | 1.34 (0.90-1.77)  p=0.000 | 7.24 (-3.84-18.32)  p=0.199 | -3.92 (-13.09-5.25)  p=0.400 | -11.16 (-22.48-0.17)  p=0.054 | 0.17 |
| Creatinine | 0.00 (-0.00-0.00)  p=0.512 | -0.00 (-0.05-0.05)  p=0.959 | -0.06 (-0.11--0.01)  p=0.026 | -0.06 (-0.11--0.00)  p=0.045 | 0.03 |
| Uric acid mg/dl | 0.01 (-0.01-0.02)  p=0.424 | 0.04 (-0.33-0.41)  p=0.834 | 0.02 (-0.35-0.38)  p=0.924 | -0.02 (-0.42-0.38)  p=0.917 | 0.00 |
| Urea mg/dl | -0.03 (-0.16-0.10)  p=0.662 | 0.20 (-2.22-2.62)  p=0.870 | -1.22 (-3.88-1.44)  p=0.366 | -1.42 (-4.17-1.32)  p=0.308 | 0.01 |
| Homocysteine mmol/l | 0.05 (-0.02-0.12)  p=0.130 | 2.68 (0.88-4.49)  p=0.004 | 1.39 (0.11-2.68)  p=0.034 | -1.29 (-3.29-0.71)  p=0.204 | 0.09 |
